# Vascular smooth muscle cells in intimal hyperplasia, an update

**DOI:** 10.3389/fphys.2022.1081881

**Published:** 2023-01-04

**Authors:** Sébastien Déglise, Clémence Bechelli, Florent Allagnat

**Affiliations:** Department of Vascular Surgery, Lausanne University Hospital, Lausanne, Switzerland

**Keywords:** peripheral artery disease, intimal hyperplasia, restenosis, vascular remodeling, vascular surgery, smooth muscle cells, neointima

## Abstract

Arterial occlusive disease is the leading cause of death in Western countries. Core contemporary therapies for this disease include angioplasties, stents, endarterectomies and bypass surgery. However, these treatments suffer from high failure rates due to re-occlusive vascular wall adaptations and restenosis. Restenosis following vascular surgery is largely due to intimal hyperplasia. Intimal hyperplasia develops in response to vessel injury, leading to inflammation, vascular smooth muscle cells dedifferentiation, migration, proliferation and secretion of extra-cellular matrix into the vessel’s innermost layer or intima. In this review, we describe the current state of knowledge on the origin and mechanisms underlying the dysregulated proliferation of vascular smooth muscle cells in intimal hyperplasia, and we present the new avenues of research targeting VSMC phenotype and proliferation.

## 1 Introduction

Intimal hyperplasia (IH) is a known complication of all types of vascular procedures, including arterial bypass, angioplasty, stenting, and endarterectomy. The progressive thickening of the vessel wall causes both an outward and an inward remodeling, leading to a narrowing of the vessel lumen, and eventually leads to impaired organ perfusion.

IH starts as a physiologic healing response to injury to the blood vessel wall (Nakano et al., 2013). As such, the process of IH is initiated by endothelial cell (EC) injury. EC constitute the interface between the blood and the vessel wall, maintaining a non-thrombogenic surface and regulating the vascular tone (vasodilation and vasoconstriction). EC loss following surgery promotes vasoconstriction, platelet aggregation and recruitment/activation of resident and circulating inflammatory cells. The “activated” EC, recruited platelets and immune cells secrete cytokines and chemokines, which trigger a pro-inflammatory response. In addition, these cells secrete growth factors, including platelet-derived growth factor (PDGF), basic fibroblast growth factor (bFGF), transforming growth factor beta 1 (TGF-β) and thromboxane A2, which stimulate a number of intracellular signaling pathways in vascular smooth muscle cells (VSMCs) and fibroblasts. Together, the secretion of these factors and the loss of EC-derived gasotransmitters nitric oxide (NO) and hydrogen sulfide (H_2_S), promote vessel remodeling and reprogramming of cells composing the media and adventitia layers. This injury-induced phenotypic modulation of VSMCs promotes repair of the lesion, but failure to resolve the healing response leads to the formation of a neointima layer between the intima and the internal elastic lamina. This new layer is made of VSMC-like cells and extracellular matrix (ECM) (Owens et al., 2004; Mylonaki et al., 2018; Chakraborty et al., 2021).

Despite decades of research and numerous clinical trials, IH remains a poorly-treated problem and a major contributor to restenosis following surgical revascularization. For open surgeries such as bypass and endarectomy, the rate of restenosis at 1 year between is 20%–30% (Simpson et al., 2014). For endovascular approaches, the rate of secondary occlusion following balloon angioplasty and stenting ranges from 30% to 60%, depending on location (Buccheri et al., 2016). IH also occurs at anastomoses in fistula creation.

## 2 Vascular smooth muscle cells

VSMCs are the most abundant cells in vessels. Located in the media layer of the vessels, VSMCs are in constant crosstalk with ECs composing the intima, resident immune cells of the vessel wall, and other signal from the ECM. Unlike skeletal muscle cells, VSMCs have remarkable plasticity, sensing, adapting and influencing other cell types and their environment (Chakraborty et al., 2021).

### 2.1 VSMC identity

In a mature blood vessel, medial VSMCs display a spindle-shaped contractile phenotype and express smooth muscle specific contractile proteins, including myosin heavy chain 11 (MYH11), calponin, smooth muscle 22α/transgelin (SM22α/tagln) and smooth muscle cell α-actin (ACTA2) (Owens et al., 2004; Chakraborty et al., 2021).

**SCHEME 1:**
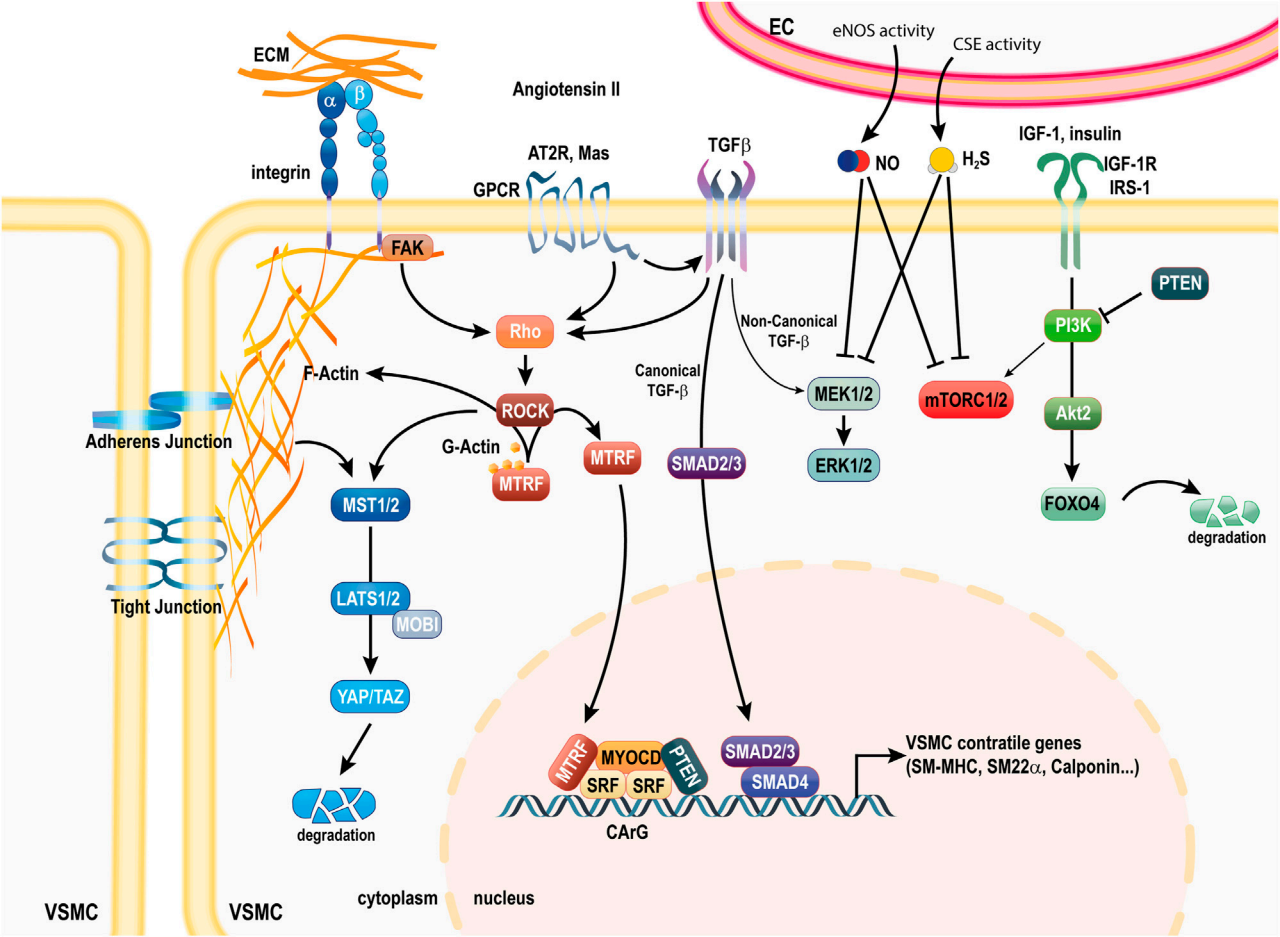
Pathways involved in the VSMC contractile phenotype. The contractile phenotype of VSMC is ensured by the coordinated activity of transcription factors SRF, MYOCD and MTRFs. Canonical TGFβ signaling through Smad2/3 promotes the activity of the SRF, MYOCD complex. YAP/TAZ degradation downstream of cytoskeleton-mediated signaling in relation to extracellular interactions with neighboring cells and the ECM maintains the contractile phenotype. FOXO4 degradation *via* Akt2 activity is also important to maintain the contractile phenotype. EC-derived NO and H_2_S ensure maintenance of the contractile phenotype by various mechanisms. PTEN also maintains the contractile phenotype *via* inhibition of PI3K activity and direct binding to SRF. Ang II and Ang-1-7 binding to the AT2R and Mas receptor potentiate the benefits of TGFβ signaling. Ang II, angiotensin II; AT2R, Ang II receptor 2; SRF, serum response factor; MYOCD, myocardin; MTRFs, myocardin-related transcription factors; FAK, focal adhesion Kinase; YAP, Yes-associated protein; TAZ, Transcriptional coactivator with PDZ-binding motif; GPCR, G protein coupled receptor; TGFβ, transforming growth factor beta; ECM, extra cellular matrix; FOXO4, Forkhead Box O4; PI3K, phosphoinositide 3-kinase; IRS1, insulin receptor 1; IGF-1R, isulin-like growth factor receptor 1; α-SMA, alpha smooth muscle actin; SM-MHC, smooth muscle myosin heavy chain; SM22α, smooth muscle 22 alpha; SMAD, Suppressor of Mothers Against Decapentaplegic 2; PI3K, phosphoinositide 3-kinase; PTEN, phosphatase and tensin homologue; NO, nitric oxide; H_2_S, hydrogen sulfide; mTORC1, mammalian target of rapamycin complex 1; MEK1/2, mitogen-activated ERK kinase; ERK1, 2, extracellular signal-regulated kinase; IGF-1, insulin like growth factor 1; IGF-1R, IGF-1 receptor; IRS1, insulin receptor 1.

The differentiated contractile identity of VSMCs is ensured at the transcriptional level *via* the serum response factor (SRF) and the VMSC-specific transcription factor myocardin (MYOCD) (Ackers-Johnson et al., 2015). SRF is a ubiquitous transcription factor binding to a general sequence motif in the CArG element (CC (A/T-rich) 6GG) to regulate the expression of marker genes (Mack and Owens, 1999). Myocardin is expressed specifically in cardiomyocytes and VSMCs, and acts as a potent coactivator of SRF and mediator of environmental cues to stimulate VSMC contractile genes (Mack and Owens, 1999; Wang et al., 2001; Wang et al., 2004). Two additional myocardin-related transcription factors (MRTF-A and B), homologous to MYOCD, form heterodimers with MYOCD to enhance transactivation of SRF (Yang and Shi, 2021). Unlike MYOCD, which is localized in the nucleus, MRTFs are sequestered in the cytoplasm through binding to G-actin monomers (Scheme 1).

### 2.2 VSMC reprogramming in IH

**SCHEME 2:**
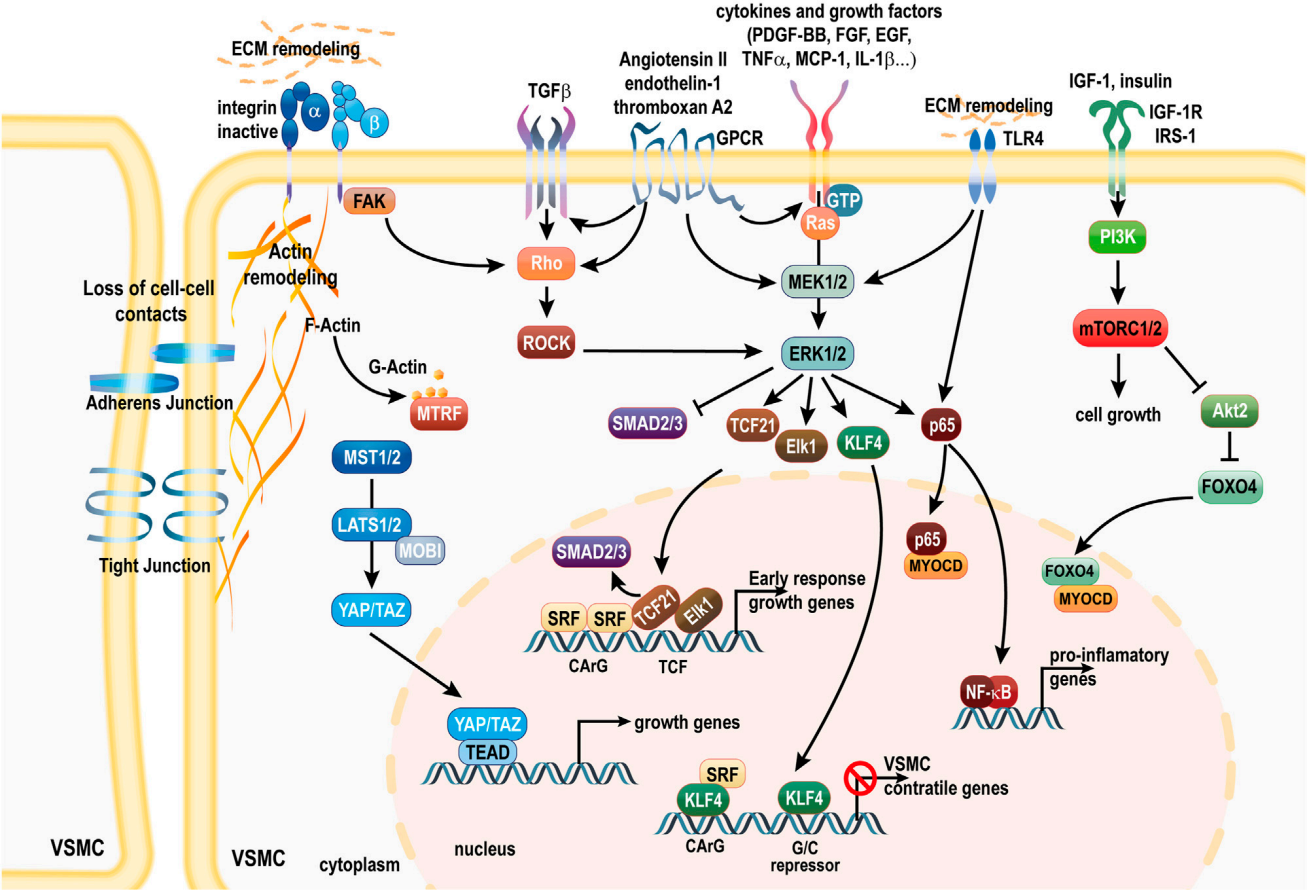
Pathways involved in the loss of the VSMC contractile phenotype. Downstream of PDGF-BB and cytokines, activation of the MAPK pathway drives disruption of the SRF/MYOCD/MTRFs complex. Non-cononical TGFβ signaling further promotes the MAPK activity and inhibition of Smad signaling. ERK mediated phosphorylation of MRTFs also prevents nuclear translocation. KLF4 and TCF members Elk1 and TCF21 displace MYOCD and induce SRF-dependent transcription of early response growth genes. mTORC1 activation promotes protein synthesis and cell growth, and Akt2 inhibition, which leads to FOXO4 translocation to the nucleus to sequester MYOCD. ECM and cell-cell interaction remodeling leads to YAP/TAZ translocation to the nucleus to promote the expression of genes associated with proliferation *via* the TEAD transcription factors. MAPK and TLR4 activation stimulates the NF-κB signaling and expression of pro-inflammatory genes. Activation of GPCR signaling *via* Ang II binding to the AT1R, thromboxane A2 or endothelin-1 binding to the ET-1R activates deleterious MAPK and ROCK signaling, and further transactivates TGFβ and growth factor signaling. AT1R, Ang II receptor 1; SRF, serum response factor MYOCD, myocardin; MTRFs, myocardin-related transcription factors; FAK, focal adhesion Kinase; YAP, Yes-associated protein; TAZ, Transcriptional coactivator with PDZ-binding motif; GPCR, G protein coupled receptor; TGFβ, transforming growth factor beta; ECM, extra cellular matrix; FOXO4, Forkhead Box O4; PI3K, phosphoinositide 3-kinase; mTORC1, mammalian target of rapamycin complex 1; KLF4, kruppel-like factor 4; TEAD, transcription enhancer activation domain; TCF21, ternary complex factor 21; Elk-1, ETS domain-containing protein-1; ET-1R, endothelin-1 receptor; ERK1/2, extracellular-signal-regulated kinase; MEK1/2, mitogen-activated ERK kinase; PDGF-BB, platelet-derived growth factor.

Unlike skeletal muscle cells, which are terminally differentiated, adult VSMC are highly plastic cells capable of profound phenotypic alterations in response to changes in their local environment (Owens et al., 2004). The ability of VSMCs to switch from a quiescent “contractile” phenotype to a proliferative “synthetic” phenotype is important for vascular injury repair. However, it also plays a complex role relevant to different pathological states, especially in the context of atherosclerosis and IH. The causal role of VSMCs plasticity in vascular remodeling during IH is undisputed (Chakraborty et al., 2021). Upon vascular injury, the concerted endothelial dysfunction and immune response modulate core transcription factors driving a gene reprogramming toward ECM production and secretion, whereas the expression of the typical VSMC markers is reduced markedly (Zhang et al., 2002; Lynch et al., 2016). VSMCs switch to a “synthetic” phenotype, characterized by a loss of contractile markers, a transition to a rhomboid morphology, and a marked increase in proliferation, migration, and protein synthesis. Matrix remodeling is driven by increased expression of proteases such as matrix metalloproteinases (MMPs), Cathepsins (Sterpetti et al., 1996; Berceli et al., 2004; Suna et al., 2018), A disintegrin and metalloproteinases (ADAMs) and ADAM with thrombospondin motifs (ADAMTSs), and matrix-associated lysyl oxidase (LOX) and tenascin (Ma et al., 2020). In addition, “activated” VSMCs exhibit a pro-inflammatory phenotype, producing tumor necrosis factors alpha (TNFα) and monocyte chemoattractant protein-1 (MCP-1/CCL2), leading to positive feedback cascade of enhanced VSMC migration and proliferation. Those synthetic VSMCs then migrate from the medial layer to the vessel intima to form a neointima layer. Growth factors, cytokines and chemokines trigger VSMCs migration and proliferation *via* the MAPK, mTOR and Hippo signaling pathways (Scheme 2). Non-coding RNAs and epigenetic modifications further modulate the activity of these pathways in the context of IH. Below is a detailed account of the role and interplay of the main pathways involved in VSMC phenotype regulation in the context of IH. Below we describe the sequence of events and various pathways involved in VSMC reprogramming in IH.

#### 2.2.1 Role of EC dysfunction

Located at the contact between the blood and the vessel wall, the EC maintain a non-thrombogenic surface and regulate the vasomotor activity (vasodilation and vasoconstriction) of vessels. In arteries, EC require high laminar shear stress to maintain proper function, i.e. secrete anti-coagulation and vasodilation agents, mainly prostacyclins and the gazotrasnmiters nitric oxide (NO) and hydrogen sulfide (H_2_S) (Stone, 2016).

Endothelial dysfunction or injury following surgery results in loss of eNOS, the enzyme producing nitric oxide (NO) and impaired H_2_S production by cystathionine γ‐lyase (CSE). NO produced and released by EC plays an important role in maintaining the quiescent contractile features of VSMC. Briefly, EC-derived eNOS-dependent NO production promotes vasodilation and VSMC relaxation *via* increased cGMP production and PKG activation to reduce cytoplasmic calcium concentration. Loss of NO also leads to expression of adhesion molecules ICAM-1, VCAM-1 and P-selectin and secretion of chemokine MCP-1, which promote platelet aggregation and leukocyte chemotaxis. NO also promotes the expression of VSMC markers and inhibits VSMC proliferation and migration *via* cGMP-dependent and independent mechanisms [Reviewed in (Cirino et al., 2017)]. The study of eNOS-deficient mice also suggest that NO deficiency promotes recruitment of stem cell antigen-1^+^ (Sca-1+)/c-Kit^−^/Lin^−^ SMC progenitor cells in a mouse model of carotid artery ligation (Zhang et al., 2006).

H_2_S works in consort with NO, often providing redundant or substituting NO in some settings (Cirino et al., 2022). Overall, the vascular effects of NO and H_2_S are mutually supporting and entangled, with both gasotransmitter having potent vasorelaxant, anti-inflammatory and anti-oxidant properties, and beneficial effect on the cardiovascular system [for full review see (Cirino et al., 2022)]. CSE expression and activity, as well as free circulating H_2_S, were reduced in patients suffering from vascular occlusive diseases (Beard and Bearden, 2011; Islam et al., 2015) and higher circulating H_2_S levels were associated with long-term survival in vascular surgery patients (Longchamp et al., 2021). Mice lacking CSE show a significant increase in IH formation as compared to WT mice in a model of carotid artery ligation (Yang et al., 2012; Macabrey et al., 2022a). On the contrary, CSE overexpression decreases IH formation in a murine model of vein graft by carotid-interposition cuff technique (Trocha et al., 2020). We and others demonstrated that several H_2_S donors inhibit IH *in vivo* in various models in rats (Meng et al., 2007), rabbits (Ma et al., 2012), mice (Yang et al., 2012; Macabrey et al., 2022a; Macabrey et al., 2022b), and in human great saphenous vein segments *ex-vivo* (Longchamp et al., 2019; Macabrey et al., 2022a; Macabrey et al., 2022b). H_2_S also directly inhibits VSMC proliferation and migration (Yang et al., 2006; Ma et al., 2012; Longchamp et al., 2019). In VSMC, H_2_S inhibits the MAPK pathway (Meng et al., 2007) and mTOR pathway (Macabrey et al., 2022a). H_2_S also limit MMP2 expression and ECMs degradation, reducing VSMCs migration (Yang et al., 2010; Yang et al., 2012). H_2_S also inhibit microtubule polymerization, leading to cell cycle arrest and inhibition of proliferation and migration in primary human VSMC (Macabrey et al., 2022b). Taken together, reduced NO and H_2_S production promotes vasoconstriction, platelet aggregation, inflammation and leucocyte infiltration and oxidative stress.

The platelets and immune cells produce and secrete growth factors including PDGF-BB, bFGF, epidermal growth factor (EGF) and TGF-β. In addition, activated ECs secrete the stromal derived factor 1α (SDF-1α), which stimulates the recruitment of progenitor cells to the vessel wall (Urbich and Dimmeler, 2004; Zhang et al., 2006; Nemenoff et al., 2011). Activated ECs also release endothelin-1, which binds to the G-protein coupled receptors (GPCR) endothelin-1 receptor and transactivate pathologic growth factor signaling including PDGF-BB, bFGF, EGF, TGF-β and thromboxane A2.

#### 2.2.2 Role of growth factors and the MAPK pathway

Originally, IH was thought to be driven by circulating cells, especially platelets, secreting platelet-derived growth factor-BB (PDGF-BB). It is now well established that VSMC proliferation is stimulated by the concerted action of several growth factors including PDGF-BB, as well as FGF, EGF and TGFβ. These growth factors mainly act *via* the mitogen-activated protein kinase (MAPK) pathways. The MAPK pathway, composed of extracellular signal-regulated kinases (ERKs), c-jun NH2-terminal kinases (JNKs), and p38MAPK, is induced by extracellular stress and regulates cell differentiation, growth and apoptosis (Muto et al., 2007). The growth factors PDGF-BB, FGF, EGF and TGFβ activate the MAPK cascade *via* the Ras/Raf/MEK/ERK pathway. Cytokines and other signals derived from oxidative stress are also strong activators of the MAPK pathway, especially JNK (Muto et al., 2007) to regulate VSMC identity (Tong et al., 2015). Overall, the MAPK pathway play a central role in VSMC proliferation and migration, and a plethora of pre-clinical studies in the last 30 years reported successful inhibition of PDGF-BB-induced-ERK or p38 activation to limit IH, including statins, a number of small inhibitor peptides, and many active compounds derived from plants providing cardiovascular benefits (Muto et al., 2007), which will not be listed here.

Downstream of PDGF-BB, ERK induce the Kruppel Like Factor 4 (KLF-4), a pluripotency transcription factor absent in contractile VSMCs. KLF4 interferes with the SRF/MYOCD module by binding to G/C repressor elements, or by competing with SRF for CArG elements to disrupt CArG–SRF–MYOCD (Deaton et al., 2009; Shankman et al., 2015). Further *in vitro* studies showed that KLF4 is required to observe PDGF-BB-induced VSMC proliferation and inhibition of MYOCD-responsive genes (Yoshida et al., 2008; Deaton et al., 2009). *In vivo*, full body Klf4 mutant mice exhibit delayed injury-induced repression of VSMC differentiation markers. However, Klf4-deficient mice displayed increased cellular proliferation in the media and IH (Yoshida et al., 2008). Therefore, the role of KFL4 in IH is likely more complex and context-dependent than *in vitro* studies suggested. SMC-specific Klf4 deletion using SM22α-Cre mice further revealed that Klf4 is required to maintain a population of Sca1^+^ progenitor VSMC in the adventitia, which may a role in adventitial remodeling upon vascular injury (Majesky et al., 2017).

MAPK activation also triggers ternary complex factors (TCFs) of the ETS-domain family, such as the ETS domain-containing protein-1 (Elk-1) (Wang et al., 2004; Yoshida et al., 2007) and TCF21 (Wirka et al., 2019; Nagao et al., 2020). These factors then displace MYOCD and induce SRF-dependent transcription of early response growth genes, leading to dedifferentiation and proliferation. ERK-mediated phosphorylation of MYOCD impairs activation of SRF and activation of VSMC contractile gene (Taurin et al., 2009). ERK-mediated phosphorylation of MRTF-A has also been shown to block its nuclear translocation in HeLa cells (Muehlich et al., 2008) and NIH3T3 fibroblasts (Panayiotou et al., 2016), which may further reduce MYOCD activity (Scheme 2).

#### 2.2.3 Role of the TGF-β non-canonical pathway

The SMAD protein family, particularly nuclear factors Smad2 and Smad3, mediate canonical TGF-β signaling. Interestingly, the canonical TGFβ signaling *via* the suppressor of mothers against decapentaplegic (SMAD) transcription factors SMAD2 and SMAD3 promotes the expression of differentiation marker SM22α, SMMHC and ACTA2, *via* enhanced binding of SRF to CArG elements within the promoters of these genes (Low et al., 2019; Cheng et al., 2022). TGFβ also stimulates the RhoA/ROCK signaling pathway and MRTFs release (O'Connor et al., 2016). However, TGF-β release in the context of endothelium injury and matrix remodeling stimulate VSMC proliferation and IH [reviewed in Low et al. (2019)]. The deleterious effect of TGF-β in the context of IH is linked to activation of the non-canonical TGFβ signaling pathway *via* the MAPK and inhibition of the SMAD signaling pathway (Kobayashi et al., 2005; Low et al., 2019). The non-canonical TGF-β signaling pathway also enhances the production and secretion of ECM protein collagen and proteoglycans in VSMCs, thus promoting the fibrosis associate with IH. In human VSMCs, thrombin or endothelin-1 binding to GPCR have been shown to transactivate the TGFβ type 1 receptor, leading to increase production and secretion of ECM protein collagen and proteoglycans (Mohamed et al., 2019). TGFβ also stimulate PDGF-B expression, amplifying PDGF-BB signaling (Low et al., 2019). Of note, SMAD3 and TCF21 may compete for the same binding site to either promote or inhibit the expression of contractile genes (Iyer et al., 2018). Thus, MAPK-induced TCF21 displace SMAD to inhibit the expression of the contractile phenotype markers. A recent study in the context of atherosclerosis using SMC-specific Smad3 deleted mice further highlight Smad3 as a key protective transcription factor again the formation of atherosclerotic plaques and vascular calcification (Cheng et al., 2022) (Scheme 2).

#### 2.2.4 Dual role of angiotensin-II signaling and GPCR singaling

Angiotensin-II (Ang-II) is the main vasoconstricting hormone and effector of the renin angiotensin aldosterone system. Ang-II drives VSMC contraction *via* binding to the type 1 Ang-II GPCR receptor (AT1R), leading to mobilization of calcium and activation of the myosin light chain kinase (MLCK) and ROCK-dependent inhibition of the myosin light chain phosphatase (MLCP). Over stimulation of the AT1R signaling in pathological conditions stimulates VSMC proliferation and hypertrophy through stimulation of the MAPK-ERK pathway (Silva et al., 2020). In addition, GPCR stimulation also transactivate growth factor receptor, including EGF receptor, PDGF receptor and FGF receptor [reviewed in Mohamed et al. (2019)].

While still controversial, Ang-II binding to the type 2 Ang-II receptor (AT2R) receptor is thought to counteract AT1R signaling. AT2R signaling maybe responsible for Ang-II-mediated stimulation of MYOCD expression and its target genes αSMA and SM-MHC, and inhibition of VSMC proliferation (Yoshida et al., 2004). Ang-II may also inhibit VSMC migration through the AT2 receptor by increasing cellular fibronectin synthesis (Chassagne et al., 2002). The anti-proliferative effect of Ang-II on VSMC might also be related to the angiotensin peptides angiotensin 1–7 (Ang-1-7). Ang-1-7 is formed by the catalytic action of ACE2 on ANG II. Ang-1-7 also counterbalances AT1R signaling, promoting vasodilation. Ang-1-7 exert its action through the GPCR Mas, and to some extent, *via* binding to the AT2R. Mas is expressed in VSMC and Ang-1-7 has been shown to inhibit VSMC migration and proliferation, and MMP expression (Silva et al., 2020). The beneficial effect of Ang-II and Ang-1–7 on VSMC phenotype also occurs indirectly, *via* ATR2- and Mas- mediated enhanced NO production in EC. Accordingly, Ang-1–7 treatment has been shown to accelerate endothelium recovery and limit IH following arterial injury (Silva et al., 2020). Overall, Ang-II has a context-dependent dual role in the modulation of VSMC phenotype, similarly to TGF-β (Schemes 1, 2).

#### 2.2.5 Role of cytokines/chemokines and the NF-κB signaling pathway

IH is associated with EC activation and inflammation. After the vascular injury, the secretion of inflammatory factors recruits inflammatory cells. Pro-inflammatory cytokines also change the structure of the extracellular matrix (ECM) to facilitate infiltration. Over the years, numerous reports demonstrated the role of various combination of chemokines and cytokines in the progression of IH, and a wide range of anti-inflammatory therapies have been proven to reduce IH in preclinical models. As mentioned earlier, cytokines and other signals derived from oxidative stress promote VSMC proliferation and migration, and IH, *via* stimulation of the MAPK pathway, especially JNK (Muto et al., 2007; Tong et al., 2015). Cytokines also stimulate the nuclear factor kappa B (NF-κB) pathway (Muto et al., 2007; Li et al., 2017). NF-κB is a master regulator of pro-inflammatory genes, including cytokines and cell adhesion molecules. Upon nuclear translocation, NF-κB (p65) directly interacts with MYOCD to inhibit the formation of the MYOCD/SRF/CArG ternary complex *in vitro* and *in vivo*, promoting the synthetic phenotype (Tang et al., 2008). Interestingly and conversely, MYOCD can also dampen NF-κB activity (Tang et al., 2008). Several studies reported that NF-κB inhibition inhibits VSMC proliferation *in vitro* (Bellas et al., 1995; Selzman et al., 1999; Sasu and Beasley, 2000) and IH *in vivo* (Zuckerbraun et al., 2003) (Scheme 2).

The pro-inflammatory cytokines TNF-α and IL-1α, secreted by macrophages/monocytes upon severe inflammation, play a central role in inflammation (Amin et al., 2020). *In vitro* studies established that IL-1α and β (Loppnow and Libby, 1990; Alexander et al., 2012; Gomez et al., 2018) and TNFα (Selzman et al., 1999; Davis et al., 2009; Lee et al., 2009) stimulate VSMC migration and proliferation. TNFα deletion prevented IH in a model of carotid artery ligation, while IL-1 type 1 receptor deletion tended to develop less IH (Rectenwald et al., 2000). Selective targeting of TNF receptors has also been shown to protect against IH (Zhang et al., 2008; Kitagaki et al., 2012; Fischer et al., 2020). The deleterious effects of TNFα may be mediated by the triggering receptor expressed on myeloid cells (TREM)-1 (Rao et al., 2016), which promotes VSMC inflammation, proliferation and migration, and is associated with in stent restenosis in patients (Wang et al., 2017).

In the context of atherosclerosis, excessive inflammation or failed inflammation resolution promotes atherosclerosis development (Back et al., 2019). Blockade of IL-1β and its receptor have been shown to limit plaque formation (Back et al., 2019; Ku et al., 2022). However, in a recent study using SMC-specific IL-1 receptor KO in ApoE^−/−^ mice, Gomez et al. demonstrated that the positive effect of IL-1β on VSMC proliferation promotes the formation of a protective SMC/collagen-rich fibrous cap during late-stage atherosclerosis (Gomez et al., 2018). Interleukin-18 blockade also inhibited IH in a rat model of vascular injury (Maffia et al., 2006). MCP-1, expressed by macrophages, ECs and VSMCs upon arterial injury, and its receptor CC chemokine receptor 2 (CCR2), are also involved in VSMC proliferation and IH in pre-clinical models (Furukawa et al., 1999; Roque et al., 2002). The inflammatory mediator toll-like receptor (TLR)-4, which signals through the MAPK and NF-κB pathway, has also been shown to contribute to IH in various animal models (Hollestelle et al., 2004; Wu et al., 2017; Rai et al., 2022). Finally, recent studies highlight the role of the NLRP3 inflammasome downstream of pro-inflammatory signals in VSMC phenotypic transformation and proliferation in hypertension (Sun et al., 2017) and atherosclerosis (Wang et al., 2018). The NLRP3-inflammasome is a large multiprotein complex activating caspase-1, which produces IL-1β and leads to cell pyroptosis. The role of NLRP3 in IH has been mostly linked to EC dysfunction and increased EC permeability. Thus, the NLRP3 inflammasome is strongly induced in EC upon pro-inflammatory exposure, and inhibition of NLRP3 inflammasome improves EC recovery and limits IH in various models (Xia et al., 2014; Koka et al., 2017; Wei et al., 2019; Li et al., 2022). Interestingly, it was recently shown that NLRP3 inflammasome activity in EC leads to horizontal transfer of IL-1β *via* extracellular vesicles, which promotes VSMC phenotypic transformation and IH (Yuan et al., 2020). Further studies are required to test whether NRLP3 inhibition in VSMC specifically would protect against IH. In contrast, the anti-inflammatory cytokines IL-10 secreted mostly by M2 macrophages, was shown to promote angiogenesis and endothelium repair, thereby resolving inflammation and reducing IH following carotid artery denudation (Verma et al., 2016).

#### 2.2.6 Role of the RhoA/ROCK module

Rock is the main effector of VSMC contraction *via* P-MLC activity (Shimokawa et al., 2016), and thus play an important role in the contractile phenotype. Mechanical forces and interactions with the ECM stimulate the expression of contractile genes *via* integrins, which activate the RhoA/ROCK signaling pathway to stimulate polymerization of G-actin monomers in filamentous (F)-actin, thereby releasing MRTFs for translocation in the nucleus (Mack et al., 2001; Miralles et al., 2003; Yang and Shi, 2021). Canonical TGFβ signaling also stimulates the RhoA/ROCK signaling pathway and MRTFs release, which enhances the transcriptional regulation of SRF and expression of the contractile gene (O'Connor et al., 2016; Mack et al., 2001; Miralles et al., 2003).

However, excessive ROCK activity downstream of GPCR activity has been shown to be involved in the deleterious vascular effects of AngII (Yamakawa et al., 2000; Higashi et al., 2003) and other GPCR ligands (Shimokawa et al., 2016). Studies *in vivo* have shown that ROCK inhibition protects from various models of IH in rats (Sawada et al., 2000; Funakoshi et al., 2001; Shibata et al., 2001) and pigs (Eto et al., 2000; Miyata et al., 2000; Matsumoto et al., 2004). Mechanistically, ROCK has been shown to promote VSMC hypercontraction and inward remodeling (Shimokawa et al., 1996; Eto et al., 2000; Kandabashi et al., 2003), VSMC proliferation and migration (Yamakawa et al., 2000; Higashi et al., 2003), and infiltration of inflammatory cells in the vessel wall (Miyata et al., 2000). In absence of myocardin or in response to mechanical strain and/or GPCR/TGFβ-activated RhoA signaling, the ROCK/SRF pathway promotes proliferation and myofibroblast differentiation (Jiang et al., 2015; Shimokawa et al., 2016; Oh et al., 2018).

Overall, the RhoA/ROCK, MAPK, and NF-κB pathways, downstream of mechanical strain and PDGF-BB, TGFβ and cytokines, integrates stress and growth signals resulting in VSMC proliferation and migration, and IH. However, no strategy based on inhibition of MAPK or NF-κB signaling limited IH in human trials (Sharma et al., 2011; Seedial et al., 2013), indicating that these pathways are not the sole responsible for VSMC proliferation and migration in IH.

#### 2.2.7 Role of the mTOR pathway

The mammalian target of rapamycin complex 1 (mTORC1) is the main hub integrating signals from the environment to control protein and nucleotide synthesis, cell growth and metabolism (Liu and Sabatini, 2020). mTORC1 is regulated *via* amino acid abundance through the GCN2 complex, and *via* glucose through the AMPK. mTORC1 activation is also under the control of growth factors, in particular insulin and the insulin-like growth factor-1 (IGF-1). Similar to insulin, IGF-1 binds to the insulin receptor or the IGF-1 receptor (IGF1R), and stimulates the phosphatidylinositol-3-phosphate kinase (PI3K)/Akt pathway and inhibit the mTORC1 repressor module TSC. Downstream of mTORC1 activation, a cascade of phosphorylation of kinases such as p70 ribosomal protein S6 kinase (p70S6K) stimulates cell growth and protein synthesis (Liu and Sabatini, 2020). In pathological condition when the MAPK is active, IGF-1 promotes VSMC proliferation and migration (Banskota et al., 1989; Bornfeldt et al., 1992; Bornfeldt et al., 1994) and IGF-1 transgenic mice display increased VSMC proliferation and migration and IH following mechanical injury (Zhu et al., 2001). Conversely, inducible IGF-1R deletion reduced the formation of neointima in a mouse model of vein graft (Cheng and Du, 2007). However, a recent study reported that the deleterious impact of IGF-1 on IH is probably mediated by binding to the insulin receptor, rather that the IGF1R (Li et al., 2019) (Scheme 2).

As the name implies, mTOR is the main target of Rapamycin, one of the two main molecules used in the clinics for the treatment of IH (see Section 3.1: current treatment of IH). Inhibition of mTORC1 by Rapamycin leads to G1-S cell cycle arrest, preventing VSMC proliferation and migration and IH (Martin et al., 2007). Forkhead box protein O4 (FoxO4) promotes VSMCs dedifferentiation by disrupting the SRF/myocardin complex (Liu et al., 2005; Jin et al., 2017). mTOR inhibits Akt2 signaling, thereby promoting nuclear translocation of FoxO4 to disrupt the SRF/myocardin complex. Inhibition of mTOR by Rapamycin rescues the VSMC phenotype (Patterson et al., 2006; Jin et al., 2017) (Scheme 1).

Recent studies also highlight a role of the late endosomal/lysosomal adaptor and MAPK and mTOR activator (LAMTOR/Ragulator) in the regulation of mTORC1 activity and IH. LAMTOR1 is a scaffold protein complex on late endosomes/lysosomes that serves as a point of convergence/integration of nutrient status and growth factor signaling. LAMTOR1 regulates mTORC1 signaling in response to amino acid concentrations (Liu and Sabatini, 2020). Liu et al. recently showed that Lamtor1 and mTORC1 signaling were significantly increased in a mouse model of arterio-venous grafting, and that SMC-specific Lamtor1 deletion prevented IH in vein grafts *in vivo* (Liu et al., 2022). In a related study, the same group demonstrated that platelet-derived microvesicles induced LAMTOR1 expression, and activated mTORC1 signaling to promote VSMC dedifferentiation in a model of mouse carotid intimal injury (Liu J. T. et al., 2021). In a recent study using inducible SMC-specific disruption of Tsc1 in mice, Li et al. showed that mTORC1 hyperactivity promoted the apparition of VSMC with a proteolytic phenotype overexpressing MMP2, leading to the formation of thoracic aorta aneurysms and dissections. These VSMC also expressed the macrophage markers Lgal3, as well as lysosomal associated membrane protein-2 (LAMP2), but not CD45, CD11b, CD68, and F4/80 (Li et al., 2020).

Recent studies also highlight a role of the phosphatase and tensin homologue (PTEN) in the regulation of VSMC phenotype and proliferation. PTEN is a lipid phosphatase working as a tumour suppressor genes *via* inhibition of the PI3K-AKT-mTOR pathway, which provides benefits against VSMC phenotype switch and proliferation (Nemenoff et al., 2008; Nemenoff et al., 2011). In addition, it was recently reported that PTEN translocate to the nucleus, where it binds to SRF to promotes SRF binding to the promoter of VSMC-specific genes such as *a*-SMA, SM-MHC and SM22α (Horita et al., 2016; Moulton et al., 2018) (scheme 1).

#### 2.2.8 Role of the hippo YAP/TAZ pathway

The Hippo pathway is emerging as a key player in VSMC proliferation. The Hippo pathway is a central regulator of early stage development in embryogenesis, vital for organ growth control and tissue homeostasis (Cai et al., 2021). The mammalian Hippo complex consist of MST1/2, LATS1/2, and MOB1, which together regulate the transcriptional co-activators Yes-associated protein (YAP) and Transcriptional coactivator with PDZ-binding motif (TAZ). The Hippo pathway senses cell density *via* tight and adherens junctions, and mechanical forces *via* integrins, FAK and Rho/Rock signaling, to regulate the transcriptional coactivators YAP/TAZ (Cai et al., 2021). YAP/TAZ also integrates signals from growth factors signaling pathway and GPCR signaling (Yu et al., 2016). The activation of the Hippo pathway leads to MST and LATS1/2 kinases activation, which phosphorylates of YAP and TAZ, leading to their degradation. When the Hippo pathway is off, active YAP/TAZ translocate to nucleus to interact with transcription enhancer activation domain (TEAD) transcription factors (TEAD1-4). The YAP/TAZ-TEAD protein complex transcribes genes that control cell proliferation and cell fate (Cai et al., 2021).

YAP/TAZ is required for vascular development but suppressed in contractile VSMC and adult cardiomyocytes (Wang et al., 2014). A recent study using inducible SMC-specific YAP/TAZ-deficient mice showed that YAP/TAZ is required to maintain the differentiated contractile phenotype (Wang L. et al., 2020). It was also recently shown that YAP/TAZ deletion results in impaired hypertension-induced vascular adaptation, leading to formation of neointimal lesions, elastin degradation and adventitial thickening (Daoud et al., 2022). Thus, YAP/TAZ is required for maintenance of vascular homeostasis. *In vitro*, overexpression of YAP or activation of YAP/TAZ by thromboxane A2 stimulated the synthetic phenotype and VSMC proliferation (Wang et al., 2012; Feng et al., 2016; Kimura et al., 2016; Huang et al., 2020). Recent evidence indicate that pulsatile laminar flow turns on the Hippo pathway, thereby targeting YAP/TAZ for degradation (Chitragari et al., 2018). In contrast, Hippo is turned off and YAP/TAZ activity upregulated in rodent models of IH where laminar flow is disturbed (Wang et al., 2012). Moreover, YAP knock-down in a rat model of carotid balloon injury, and SMC-specific YAP deletion in a mouse model of carotid artery ligation, reduced injury-induced VSMC phenotypic switch and IH (Wang et al., 2012). Another recent study highlights the key role of YAP downstream of FAK and Rho/ROCK signaling in the deleterious effect of Osteoprotegerin, a secreted protein involved in atherosclerosis, vascular calcification and matrix degradation (He et al., 2020).

TEAD1 is also induced after vascular injury, and SMC-specific TEAD1 deletion inhibits IH in mice (Osman et al., 2019). Interestingly, in this study, they report that TEAD1 promotes mTORC1 activation (Osman et al., 2019). Studies in the context of cancer also highlight a cross talk between MRTF/SRF and YAP-TEAD to regulate invasion (Foster et al., 2017; Kim et al., 2017). Additional recent studies suggest further cross-talk between the Hippo and mTORC1 pathways *via* microRNAs and regulation of autophagy to control cell growth and proliferation [reviewed in Ostriker and Martin (2019)]. Overall, the YAP/TAZ-TEAD module seems to play a central role in vascular function and adaptation, and dysregulation of this pathway contributes to IH in several ways.

#### 2.2.9 Modulation by non-coding RNA

A large number of studies also support a major role of non-coding RNAs in the regulation of VSMC phenotype. Non-coding RNAs interact with DNA, proteins, and other RNA molecules, thus acting as versatile modulators of major cellular processes. Thus, microRNA (miRNAs), long noncoding RNA (lncRNAs) and circular RNAs (circRNAs) expressed in VSMCs have been described to control VSMC phenotype switching, proliferation, migration and apoptosis. In this review, we will not list all the non-coding RNA that have been described to play a role in VSMCs. For this, we direct the reader to recent reviews focused only on non-coding RNAs (Leeper and Maegdefessel, 2018; Maguire and Xiao, 2020).

Non-coding RNAs have been described to either protect or contribute to IH, depending on their profile of expression. Thus, non-coding RNAs that are absent in contractile VSMC but overexpressed in synthetic VSMC (miR-21, miR-146a, lnc NEAT-1) tend to promote the synthetic phenotype. Conversely, non-coding RNAs that are down-regulated in IH (miR-22, miR-24, miR-143/miR-145, miR-663, lnc GAS5) tend to promote VSMC differentiation when overexpressed. Many non-coding RNAs have been reported to modulate the MYOCD/SRF module, KLF4 and FOXO4. Thus, down-regulated non-coding RNA such as the miR-143/miR-145 cluster could play an important in maintenance of the contractile phenotype *via* modulation of KLF4 and MYOCD/SRF (Leeper and Maegdefessel, 2018; Maguire and Xiao, 2020). Additional recent studies indicate that microRNAs regulate interactions between the Hippo and mTORC1 pathways to control cell growth and proliferation [reviewed in (Ostriker and Martin, 2019)].

Non-coding RNAs also regulate VSMC apoptosis and survival pathways. Several miRNAs regulate PTEN expression in VSMCs, thereby influencing the PI3K/Akt/mTOR pathway (miR-26a, miR-21), cell proliferation and survival (Horita et al., 2011; Lin et al., 2021). Non-coding RNAs also regulate caspase activation, either through Bcl2-mediated regulation (miR-21, HIF1α-AS1), or through the tumor suppressor p53 (Linc-p21, circ-ANRIL, H19).

Other non-coding RNAs such as miR-29a/b/c, miR-195 and long non-coding RNA HAS-AS1 have been described to regulate ECM production and matrix remodeling. Recent evidence also suggest that miR-155-5p inhibition *via* STAT3 facilitates reticulocalbin 2-driven vascular calcification (Zhao et al., 2021). Of importance, several miRNAs and long-non coding RNAs have been described to contribute to VSMC to VSMC cross-talk and communication between VSMC and EC or VSMC and platelets through horizontal transfer of non-coding RNAs *via* microvesicles or exosomes (Zeng et al., 2019).

Many non-coding RNAs have been implicated in VSMC biology. However, many of these non-coding RNAs are not specific to VSMCs, and may have different roles in other tissues and pathologies (Leeper and Maegdefessel, 2018; Maguire and Xiao, 2020). Dong et al. recently identified *CARMN* (cardiac mesoderm enhancer-associated noncoding RNA) as a highly abundant SMC-specific lncRNA downregulated in various vascular diseases. *CARMN* was further demonstrated to maintain the VSMC contractile phenotype both *in vitro* and *in vivo* by directly binding to MYOCD and potentiating MYOCD function (Dong et al., 2021).

The pleiotropic effects of non-coding RNA, together with their mobility and non-cell specificity, may limit their therapeutic potential. That said, they play a central role in response to pharmacological treatment and future strategies targeting VSMC phenotype and proliferation will need to take into account this complex network playing a key role in gene regulation.

#### 2.2.10 Epigenetic modulation of VSMC identity

Gene expression is regulated at the chromatin level by epigenetic regulation, which refers to modifications in DNA or histones that shift chromatin accessibility. These include DNA methylation and post-translational modifications of histones (acetylation, methylation, phosphorylation, etc.). Recent evidence suggest that the phenotype of VSMC is also controlled *via* epigenetic modifications.

##### 2.2.10.1 DNA methylation

DNA methylation is associated with chromatin condensation and gene repression. Once though irreversible and a sign of terminal cell differentiation, DNA methylation is now recognized as a dynamic process of *de novo* methylation, maintenance of the methylated cytosine, and demethylation. The DNA methyltransferase family (DNMTs) catalyzes DNA methylation. DNMT3A and DNMT3B are responsible for the *de novo* methylation. DNMT1 maintains DNA methylation pattern through cell replication (Lyko, 2018). DNA demethylation occurs both passively and actively *via* the ten-eleven translocation methylcytosine dioxygenase (TET) (Lyko, 2018).

In 2013, Liu et al. showed that TET2 knockdown inhibits expression of MYOCD and SRF, with concomitant upregulation of KLF4, while TET2 overexpression was sufficient to induce a contractile phenotype (Liu et al., 2013). They further show that local viral-mediated Tet2 overexpression or knock-down at the site of injury in a mouse model of femoral wire injury reduced or increased IH, respectively (Liu et al., 2013). The authors propose TET2 as a master epigenetic regulator of VSMC differentiation. Interestingly, TET2 knockdown prevented rapamycin-induced VSMC differentiation, suggesting an interplay with mTORC1 (Liu et al., 2013).

It was also shown that DNMT-1, the key DNA methyltransferase maintaining DNA methylation pattern, methylates and suppress the TET2 gene in VSMCs, thereby preventing TET2-mediated contractile gene demethylation (Zhuang et al., 2017). It was also recently shown that TET2 expression is under the control of non-coding RNA miR-22-3p and circMap3k5 (Zeng et al., 2021). Furthermore, Jeong et al. discovered that FAK, which is induced upon vascular injury, elicits VSMC dedifferentiation by stabilizing DNMT3A. They further show that FAK inhibition leads to DNMT3A degradation and DNA hypomethylation of contractile gene promoters, which increased VSMC contractile protein expression (Jeong et al., 2021).

##### 2.2.10.2 Histone acetylation

Histone acetylation is another epigenetic modification that opens the chromatin to facilitate transcription. Histone acetylation in governed by histone acetyltransferases (HATs) such as p300 and CREB-binding protein (CBP), which promote chromatin opening. Concersely, Histone deacetylases (HDACs) remove acetyl groups from lysine residues to close chromatin. HDAC hyperactivity is a hallmark of cancer and promotes cell proliferation. Recently, Chakraborty et al., demonstrated, using VSMC-specific knockout mice, that p300 and TET2 are mutually required to stimulate the expression of contractile markers, while CBP facilitates the recruitment of HDAC2 and 5 to contractile protein promoters to lock the chromatin (Chakraborty et al., 2022).

Downstream of PDGF-BB, KLF4 may associate with HDAC2 on contractile gene promoters to repress their expression (McDonald et al., 2006; Yoshida et al., 2008). PDGF-BB also increases HDAC4 expression and activity, and HDAC4 knockdown inhibits VSMCs proliferation and migration (Usui et al., 2014). In addition, HDACs have been describe to regulate non-histone proteins, including myocardin/MRTF, SRF, and KLF4. Thus, HDAC4 and HDAC5 suppress VSMC contractile gene expression *via* binding to MYOCD (Cao et al., 2005). HDAC6 sequesters MRTF-A in the cytosol, which facilitates PDGF-BB-induced repression of contractile genes (Yoshida et al., 2007; Zhang et al., 2018).

##### 2.2.10.3 Histone methylation

Gomez et al., showed that dimethylation of lysine 4 of histone H3 (H3K4me2) at the MYH11 locus is a hallmark of VSMC in human and mouse, which is kept even in atherosclerotic dedifferentiated VSMC with no detectable expression of VSMC marker genes (Gomez et al., 2013). Another recent study further uncovered that H3K4me2 removal in VSMC is sufficient to induce the synthetic phenotype, due to impaired recruitment of TET2, leading to loss of miR-145 expression. Consequently, *in vivo* editing of H3K4me2 exacerbates VSMC plasticity and IH (Liu M. et al., 2021).

### 2.3 The origins and lineage fates of neointimal cells

#### 2.3.1 Contribution of VSMC

IH is probably, for the main part, formed by proliferating VSMC originating from dedifferentiated contractile medial VSMC. However, it is now well accepted that arterial and neointimal VSMCs are phenotypically heterogeneous and the origin and identity of the VSMC composing the neointima remains debated (Allahverdian et al., 2018; Chakraborty et al., 2021). Recent VSMC lineage tracing studies using *in vivo* cell fate tracing with SMC-specific genetic reporter tools suggest that previous single marker-based studies might have failed to identify VSMCs correctly. These new studies also question the multiple origin of neointimal cells, advocating for VSMC-derived multiple cell types present in atherosclerotic and neointima lesions. Multicolor lineage tracing further suggest that a small subset of VSMCs expand after injury to form clonal patches of neointimal cells (Chappell et al., 2016; Wang Y. et al., 2020; Worssam et al., 2022). Further studies are required to elucidate the identity of this small subset of VSMCs. That said, a large body of literature still describes that neointimal may arise from other local or circulating cells.

#### 2.3.2 Contribution of adventitial fibroblasts

In addition to medial VSMC-derived cells, neointimal-cells have been documented to have various origins. The most abundant, after medial VSMC, are probably myofibroblasts. Myofibroblasts originate from quiescent fibroblasts, the most common cell type in the adventitia, which have converted into proliferating fibroblasts expressing several VSMC markers such as α-SMA, SM-22α and calponin. These cells migrate into the media-neointima layer where they secrete pro-inflammatory cytokines, chemokines and altered ECM and metalloproteinase components (Sartore et al., 2001; Tinajero and Gotlieb, 2019). On the other hand, several studies in atherosclerosis using EC-lineage tracing have shown that endothelial-to-mesenchymal transitions (EndoMT) account for a large fraction of MSCs evolving into myofibroblasts. Thus, 20%–45% of myofibroblasts in atherosclerotic lesions would be of endothelial origin, and 20% of all EC express ACTA2 in ApoE^−/−^ under high cholesterol diet (Chen et al., 2015; Evrard et al., 2016). EndoMT may play an important role in atherosclerosis but the role and extend of EndoMT in IH remains uncertain. Further recent lineage study (see Section 2.3.4) question the origin of neointimal myofibroblasts.

#### 2.3.3 Contribution of progenitor cells

Accumulating evidence also shows that neointimal cells may come from progenitor cells originating from the vessel wall, especially the adventitia layer (Wang et al., 2015; Roostalu et al., 2018). These studies support the existence of various populations of mesenchymal stem cells (MSCs) or multipotent vascular progenitor cells within the vessel wall. In 2004, Hu et al. were the first to characterize progenitor cells positive for Sca-1, c-kit and CD34 in the adventitia layer of ApoE^−/−^ mice, and to demonstrate that these cells could differentiate into myofibroblasts and VSMC found in the intima layer of atherosclerotic lesion (Hu et al., 2004). Further studies identified similar progenitor cell populations in human arterial and venous tissue (Torsney et al., 2007; Campagnolo et al., 2010; Klein et al., 2011), suggesting a role for these cells in arterial remodeling and IH. In contrast, recent evidence suggest that Sca1 upregulation is a hallmark of VSMCs undergoing phenotypic switching in atherosclerotic plaques (Dobnikar et al., 2018).

The cells composing the neointima may also arise from circulating progenitor cells or from the bone marrow (Shimizu et al., 2001). However, the contribution of circulating progenitor cells to IH seems to depend upon the model and the type of injury. In 2003, Tanaka et al. demonstrated that bone marrow cells contribute up to 50% of VSMC in the neointima in a model of wire-mediated endovascular injury (Tanaka et al., 2003). In contrast, they observed only a few bone marrow-derived cells in the neointima in a model of carotid artery ligation and almost no detectable cells in a model of perivascular cuff replacement (Tanaka et al., 2003). Similarly, studies in the atherosclerosis field yield conflicting results regarding the role of bone marrow derived cells in atherosclerotic VSMC (reviewed in details in [Albiero et al., 2010; Gori, 2022)]. Studies in human in the context of atherosclerosis using cross-sex bone marrow transplant also identify that 10%–20% of VSMC marker-positive cells in coronary artery lesions are of myeloid origin (Caplice et al., 2003; Iwata et al., 2010). However, most neointimal cells are likely of medial origin, as demonstrated with *ex vivo* studies using human vessels showing that IH forms in a vessel self-sufficient manner, independently of circulating factors or cells (Prandi et al., 2015; Longchamp et al., 2019). Circulating progenitor and bone marrow-derived cells probably play a more important role in the endothelium repair (Griese et al., 2003; Hagensen et al., 2012; Wang et al., 2021; Gori, 2022) than through direct contribution to the VSMCs composing the neointima. That said, a recent study using cell fate mapping and single-cell RNA sequencing identified Sca1^+^ vascular progenitors in the adventitial layer of artery walls. The authors show that these cells migrate into the medial layer where they proliferate as *de novo* VSMCs faster than medial VSMCs (Tang et al., 2020). Drawing conclusions from these studies remains challenging given the small number of studies and the variety of experimental models and methodology, especially the methods and markers employed to isolate and identify cell types.

#### 2.3.4 Recent insight from lineage tracing studies

Recent VSMC lineage tracing studies using *in vivo* cell fate tracing with SMC-specific genetic reporter tools suggest that previous single marker-based studies might have failed to identify VSMCs correctly. Even though many classical VSMC markers such as αSMA and SM22α are problematic (Sui et al., 2014; Chakraborty et al., 2019), MYH11 may remain a stable VSMC protein still present in VSMC-derived neointimal cells (Xia et al., 2014; Islam et al., 2015). Lineage tracing of bone marrow-derived progenitors in a model of femoral wire-induced injury revealed that circulating progenitors are recruited to injured vessels but do not differentiate into VSMC, but mostly to macrophages (Iwata et al., 2010; Nemenoff et al., 2011). Similarly, using a model carotid artery ligation, Herring et al. found that 80% of the neointimal cells derive from Myh11^+^ or Acta2^+^ cells (Herring et al., 2014). Additional studies in the context of atherosclerosis reported that 30%–70% of plaque cells originate from VSMC (Shankman et al., 2015; Chappell et al., 2016), while up to 80% of VSMC-derived cells in the plaques do not express the VMSC markers Acta2 (Gomez et al., 2013; Shankman et al., 2015). It was further observed that about 7% of VSMC-derived cells are Sca1^+^ mesenchymal stem cells (MSCs) and 12% are Acat2^+^ Pdgfβr^+^ myofibroblasts-like cells, accounting for about 50% of the myofibroblasts-like cells found in the plaque (Shankman et al., 2015). A recent study also highlights that neointimal cells arise from a small number of clonal Sca1^+^ dedifferentiated VSMC (Worssam et al., 2022). These studies question the fact that myofibroblasts arise from adventitial fibroblasts. Further lineage tracing studies will be required to assess the contribution of medial VSMC to the population of myofibroblasts in the context of IH. Similarly, it seems that Sca1^+^ cells, usually referred to as MSCs, may have various origins. Originally, it was proposed that MSCs come from medial or adventitial resident or circulating progenitor cells, but it is now clear that some MSCs cells are dedifferentiated medial VSMC expressing the stemness marker Sca1 (Dobnikar et al., 2018). In contrast, myeloid-derived cells have be shown to express VSMCs markers, such as Sm22α and Acta2 in the context of atherosclerosis (Sata et al., 2002; Caplice et al., 2003).

##### 2.3.4.1 VSMC-derived neointimal cells may have a osteo-chondrogenic phenotype

In the context of atherosclerosis, lineage tracing identified VSMC expressing the macrophage markers CD68 and/or Lgals3, suggesting that some VSMCs may dedifferentiate in macrophage-like cells. However, recent single-cell RNA sequencing from murine atherosclerotic lesions in ApoE^−/−^ with VSMC lineage tracing using Tagln and calponin suggest that Lgals3^+^ may be an early marker of phenotypic modulation towards fibroblast-like cells, which they term “fibromyocytes”, rather than into classical macrophages (Wirka et al., 2019). Using a similar approach, Alancar et al. also observed that Lgals3 is a marker of an early transitional state of VSMCs with an ECM remodeling phenotype, which ultimately contribute to three populations of osteogenic and other pro-inflammatory non-macrophage VSMCs-derived cells (Alencar et al., 2020). Other recent studies describe this intermediate multipotent cell type during atherosclerosis (Pan et al., 2020; Hartmann et al., 2021), which could differentiate into inflammatory cells and fibro/osteochondrogenic cells, as well as return toward the VSMC phenotype (Pan et al., 2020; Hartmann et al., 2021). Another recent study identified five VSMC-derived cell populations among CD45^−^ cells in the atherosclerotic aorta of ApoE^−/−^ under high cholesterol diet. Based on their gene expression profile, these were labelled macrophagic/calcific phenotype, mesenchymal/chondrogenic phenotype, inflammatory/fibro-phenotype and inflammatory phenotype (Brandt et al., 2022). Of note, these cells all express KLF4, the main MYOCD/SRF disruptor (Wirka et al., 2019; Alencar et al., 2020; Pan et al., 2020) and SMC-specific Klf4 knockout leads to marked reduction in Lgals3^+^ VSMC and reduced atherosclerotic plaques (Shankman et al., 2015; Alencar et al., 2020). Thus, Lgals3 might be a marker of stemness rather than a marker of macrophages. That said, some VSMC-derived cells do express traditional macrophage markers CD11b and F4/80 (Dobnikar et al., 2018; Alencar et al., 2020), and have been shown to perform nonprofessional phagocytosis and contribute to the population of pro-inflammatory foam cells in atherosclerotic plaques (Vengrenyuk et al., 2015; Wang et al., 2019). Among all these studies conducted in the context of atherosclerosis, one study was performed after partial carotid artery ligation in the mouse. This study identified 15 clusters 1-week post injury, among which four EC-derived cell populations involved in lipid metabolism and lipid storage, mechanotransduction or undergoing EndoMT transition (Li et al., 2021). Of note, the study identified an intermediate VSMC population progressing into fibro/osteochondrogenic-like VSMCs. Pro-inflammatory cell were all of CD45^+^ origin (Li et al., 2021). Osteo-chondrogenic differentiation of VSMCs contributes to vascular calcification in vascular diseases (Abbasian, 2021) and this osteo-chondrogenic gene signature suggest that VSMC may transition towards osteoblast-like cells leading to vascular calcification. Vascular calcification plays a major role in arterial stiffness in peripheral artery disease, as well as in atherosclerosis, chronic kidney disease, hypertension, and diabetes (Durham et al., 2018; Yu and Li, 2020). Vascular calcification begins as microcalcification near the internal elastic lamina, which progresses to calcified nodules. Reactive oxygen species and inflammatory mediators in the vessel wall, such as TNF-α, increase the expression of Msx2, which increases the expression of Runt-related transcription factor 2 (RUNX2), SOX9 and osterix (Speer et al., 2009; Bostrom et al., 2011; Lin et al., 2016). These transcription factors upregulate osteogenic markers such as osteopontin, osteocalcin, bone morphogenetic protein-2 (BMP-2), and alkaline phosphatase. Recent studies highlight a key role of VSMC-VSMC cross-talk in vessel calcification, *via* the release of exosomes carrying cargo such as mRNAs, miRNAs and peptides regulating the expression of osteogenic markers such as RUNX2 in the recipient cells (Wu et al., 2022). Of note, Gli1^+^ mesenchymal stem cells and circulating stem cells may also differentiate into osteoblast-like cells, and play a role in vessel calcification (Demer and Tintut, 2008; Toth et al., 2020; Yu and Li, 2020).

**SCHEME 3:**
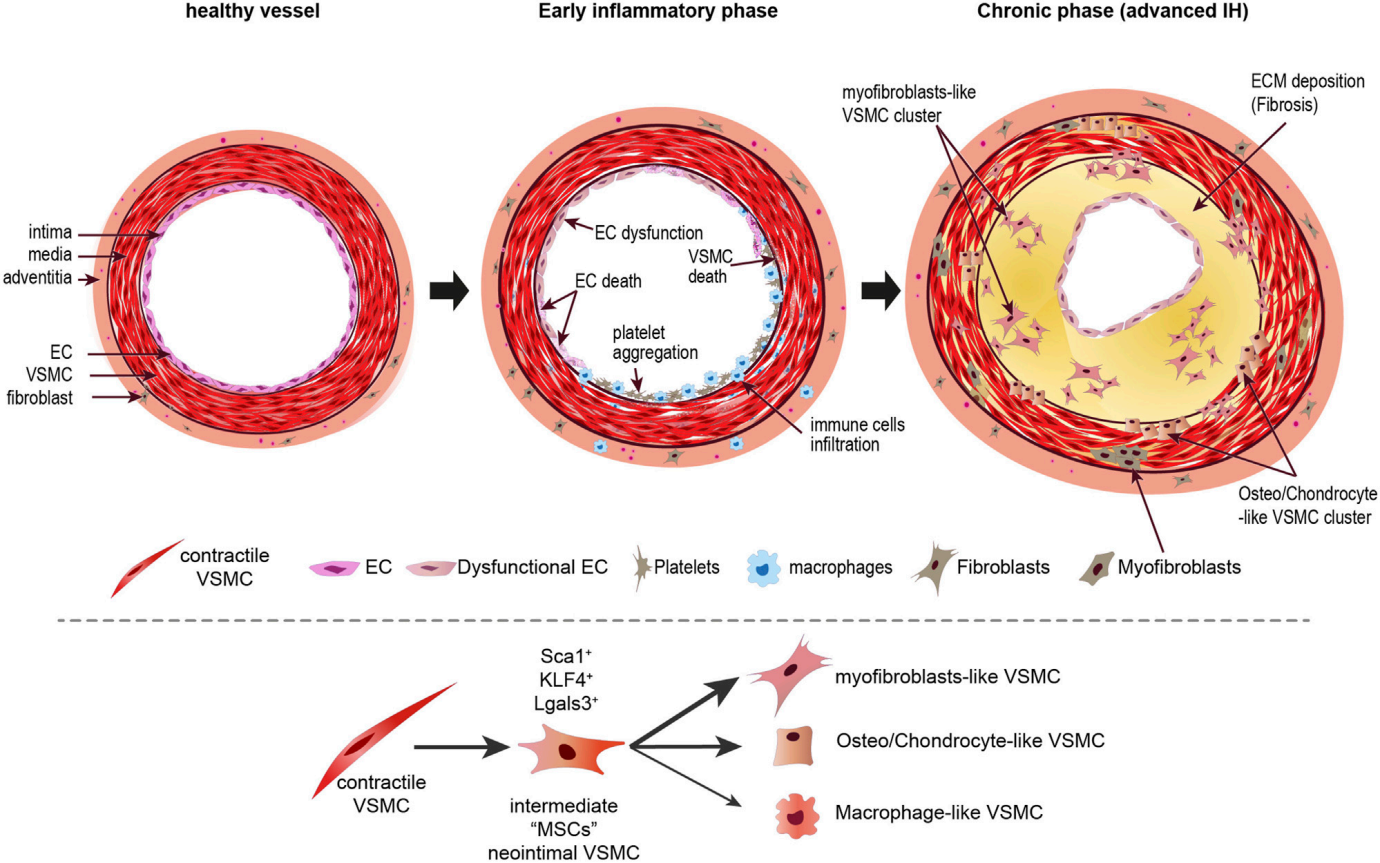
Phenotypic transition of VSMCs in intimal hyperplasia. Upon vessel injury, EC dysfunction and death triggers an early inflammatory response leading to recruitment of platelets and immune cells, which secrete factors facilitating reprogramming of VSMC toward proliferating and secreting VSMCs. Recent evidence suggest that a few VSMC first transition to an intermediate MSC-like phenotype before clonal expansion of clusters of cells secreting ECM components and osteo and chondrocyte markers. While important in the context of atherosclerosis, it is unclear whether VSMC transdifferentiate in pro-inflammatory macrophage-like cells during IH. Additionally, myofibroblasts probably arise from adventitial fibroblast and resident or circulating progenitor cells. EC, endothelial cells; VSMC, vascular smooth muscle cells, MSC, mesenchymal stem cells; Sca1, Stem cells antigen-1 (Ly6a); Klf4, kruppel-like factor 4; Lgals3, galectin 3.

It should be noted that no study described macrophage-like VSMCs in the context of IH and studies suggest that inflammatory cells in IH mainly come from circulating CD45^+^ cells (Iwata et al., 2010; Nemenoff et al., 2011; Chappell et al., 2016; Li et al., 2021). It is unlikely that macrophage-like VSMC arise in neointimal lesion given that inflammation is transient in IH and IH lesion do not feature foam cells. New spatial transcriptomics techniques will also bring new understanding into the spatiotemporal regulation of VSMC fate, clonality, differentiation, and phenotypic modulation in the context of IH (Scheme 3).

##### 2.3.4.2 Which pathways drive the osteo-chondrogenic phenotype?

Related to Section 2.2 of this review and the triggers of differentiation trajectories, current studies highlight a key of KLF4 upstream of Lgals3^+^ (Shankman et al., 2015; Alencar et al., 2020). However, further studies will be required to determine the role and contribution of the MAPK, TGFβ, NF-κB, mTOR and YAP/TAZ pathways to the formation of different population of neointima VSMC-derived cells. The recent lineage studies underscore the enrichment of osteo-chondrogenic-like VSMCs in neointimal lesions. The canonical Wnt/β-catenin signaling pathway play a key role in osteogenesis and it has been shown to modulate Runx2 expression and VSMC osteogenic transdifferentiation and calcification (Cai et al., 2016; Tian et al., 2019; Voelkl et al., 2019; Huang et al., 2022). The TGF-β/BMP/SMAD pathway also regulate MSC differentiation during skeletal development, bone formation and bone homeostasis (Wu et al., 2016). However, its role as inducer of osteo-chondrogenic differentiation in VSMCs is more controversial. Thus, BMP2 stimulated osteogenic VSMCs differentiation in ApoE^−/−^ mice (Nakagawa et al., 2010), whereas BMP7 protected against vascular calcification in LDLR^−/−^ mice (Mathew et al., 2006). TGF-β promoted the chondrogenic phenotype in a mouse model of calcification *via* matrix Gla protein deletion (Beazley et al., 2015). Conversely, SMC-specific deletion of TGF-β receptor 2 resulted in VSMC transdifferentiation into an MSC-like intermediate state that generated osteoblasts, chondrocytes, adipocytes, and macrophages in Apoe^−/−^ mice (Chen et al., 2020). Of note, inflammation and the NF-κB signaling pathway have also been reported to drive osteogenic VSMCs differentiation in various models (Zhao et al., 2012; Zhou et al., 2014; Yoshida et al., 2017; Voelkl et al., 2018; Lee et al., 2019; Voelkl et al., 2019). In recent years, increasing evidence also suggest that mTOR plays important roles in the differentiation of mesenchymal stem cells (MSCs) into osteoblasts and chondrocytes (Cai et al., 2022). Thus, it is likely that mTOR pathways contribute to the formation VSMC-derived MSC and subsequent formation of myofibroblasts and osteo-chondrogenic-like VSMCs in the context of IH. In a recent study using inducible SMC-specific disruption of Tsc1 in mice, Li et al. showed that mTORC1 hyperactivity promoted the apparition of VSMC with a proteolytic phenotype overexpressing MMP2, leading to the formation of thoracic aorta aneurysms and dissections. These VSMC also expressed the macrophage/stemness marker Lgal3 (Li et al., 2020). Finally, the study of inducible SMC-specific YAP/TAZ-deficient mice showed that cytoplasmic YAP/TAZ inhibit nuclear translocation of Disheveled 3 (DVL3), which drives osteogenic transdifferentiation of VSMCs (Wang L. et al., 2020).

##### 2.3.4.3 Limitations and future works

These new evidences underscore how little is known about the identity and origin of the cells responsible for the formation of IH. Recent single-cell RNA sequencing combined with VSMC lineage tracing led to new insights into VSMC phenotypic switching and evolution in the context of atherosclerosis. So far, these new techniques have been seldom used in IH models. Similar, but definitely different, differentiation trajectories probably occur during IH, with an enrichment in myofibroblasts and osteo-chondrogenic-like VSMC. Future studies using various models and human tissues will probably uncover more phenotype variations. The advent of new genetic tools has allowed inducible SMC-specific CRE recombination and VSMC tracing. However, all current promoters results in recombination in both vascular and visceral SMC lineages (Chakraborty et al., 2019), which often lead to visceral myopathies (Angstenberger et al., 2007; Huang et al., 2015; Daoud et al., 2022). New Cre lines targeting VSMC-only would be useful to understand further the biology of VSMCs.

Several studies underscore that the origin of neointimal cells varies depending on the model. Thus, Roostalu et al. showed that in a model of wire-induced arterial injury, medial VSMCs were the primary contributors to IH. In contrast, supermicroanastomosis of the femoral artery around a nylon monofilament used as a stent resulted in early smooth muscle death and subsequent colonization of the vascular wall by adventitial cells and IH (Roostalu et al., 2018). Tanaka et al. also demonstrated that bone marrow cells contribute up to 50% of VSMC in the neointima in a model of wire-mediated endovascular injury, whereas only a few bone marrow-derived cells were found in the neointima in a model of carotid artery ligation, and almost no detectable cells in a model of perivascular cuff placement (Tanaka et al., 2003). In a recent study, Tang et al. also showed, in the context of femoral wire injury, that adventitial Sca1^+^ progenitor cells play an important role in VSMC expansion (Tang et al., 2020). These studies highlight that the origin and composition of IH probably differs between models and vascular bed, and depends on the level of damage to the media layer.

Of note, all cell-lineage evidence arise from mouse models of arterial injury whereas IH is strikingly different in rodent models and humans. In mouse models, IH develops as a VSMC-rich neointima with high proliferation rates in both the media and the neointima layers, and little ECM deposition (Perkins, 2010; Allagnat et al., 2016; Allagnat et al., 2017). In contrast, IH *ex vivo* in human vein segments features extensive ECM remodeling and collagen deposition, accompanied by VSMC apoptosis and low VSMC proliferation (Longchamp et al., 2014a; Longchamp et al., 2014b; Longchamp et al., 2019; Macabrey et al., 2022b). This morphology is more reminiscent of the lesions observed in patients who developed rapid restenosis following angioplasty or stent placement (Farb et al., 2004; Nakano et al., 2013). These fundamental differences may explain, in part, why strategies targeting VSMC proliferation were successful to limit IH in pre-clinical models, but failed in human clinical trials. These differences may also explain the controversies regarding the origin, identity and role of the cells composing the neointima. Overall, the origin and nature of neointimal cells remains unclear and probably differs in mouse vs. human, in large vs. small arteries, and in venous graft vs. arterial injury.

## 3 Treatment of intimal hyperplasia

In this section, we review the current agents targeting VSMC proliferation and IH, their limitations, and new avenues of research aimed at VSMC-proliferation.

### 3.1 Current treatments

Numerous drugs have been tested over the years to limit restenosis. However, in most trials, the use of systemic drug therapy to prevent restenosis failed, due either to poor tolerance or lack of efficacy (Sharma et al., 2011; Seedial et al., 2013). The catheter-based endovascular interventions are taking advantage of the focal nature of atherosclerotic lesion and the plain old balloon angioplasties (POBA) and bare metal stents (BMS) strategies have revolutionized the management of vascular occlusive diseases. However, these devices suffered from high rates of in-stent restenosis (ISR) due to IH. To circumvent this problem, drug-coated balloons (DCB) and drug-eluting stents (DES) have been developed to reduce restenosis using local drug administration, which allows delivery of higher doses of drugs while minimizing systemic side effects. These medical devices are now the treatment of choice for endovascular approaches to treat short lesions in coronary or femoral arteries.

The most used drug is the anti-tumor chemotherapy Paclitaxel (Taxol™), a chemotherapeutic agent that stabilizes microtubule assembly by binding β-tubulin dimers, preventing their depolymerization. The low doses of paclitaxel in DES induce a cytostatic G1 cell cycle arrest, inhibiting proliferation and migration without inducing apoptosis. Several paclitaxel-coated balloons and eluting stents with various formulations and doses of paclitaxel demonstrated superiority to POBA (Caradu et al., 2019; Teichgraber et al., 2020; Abdoli et al., 2021) or BMS (Ding et al., 2018; Abdoli et al., 2021).

Another drug used in DCB and DES is Rapamycin, also known as Sirolimus. Rapamycin inhibits the mammalian target of rapamycin complex 1 (mTORC1), a cellular sensor of amino acid abundance and growth factor signaling. mTORC1 is the main hub integrating signals form the environment to control protein and nucleotide synthesis, cell growth and metabolism, as well as proliferation and migration (Liu and Sabatini, 2020). Inhibition of mTORC1 by Rapamycin leads to G1-S cell cycle arrest, preventing VSMC proliferation and migration, and IH (Martin et al., 2007). In addition, Rapamycin promotes VSMC differentiation *via* Akt2 signaling, which drives FoxO4 export from the nucleus. Akt2 activation is also antiapoptotic and improves insulin sensitivity (Patterson et al., 2006; Jin et al., 2017). Rapamycin also induces the master epigenetic regulator TET2 to stimulate VSMC differentiation (Liu et al., 2013). The pleiotropic effects of rapamycin on VSMC explains its efficacy and DES coated with Sirolimus and its analogs everolimus and zotarolimus are currently the preferred choice for coronary revascularization (Kaul et al., 2015; Byrne et al., 2017; Teichgraber et al., 2021).

### 3.2 Limitations of current therapies

Although the rapamycin- and paclitaxel-eluting stents have improved outcomes compared with POBA and BMS, challenges remain. Overall, the arrival of DES and DCB reduced the incidence of restenosis below 10% in coronary arteries (Fattori and Piva, 2003), but restenosis has been delayed rather than suppressed (Jukema et al., 2011). DES also require prolonged antiplatelet therapy and hinder future surgical revascularization. In addition, the endovascular treatment of peripheral artery disease using DES is more complicated as rates of ISR after femoropopliteal artery stenting still range between 20% and 40% at 1 year (Aru and Tyagi, 2022). In peripheral *below the knee* small arteries, the use of DCB is controversial, and stents are not recommended due to the risk of thrombosis (Bjorck et al., 2020). Recently, various systematic review and meta-analysis questioned the widespread use of paclitaxel for the treatment of restenosis (Beckman and White, 2019). Indeed, conflicting analysis identified (Katsanos et al., 2018; Rocha-Singh et al., 2020; Royce et al., 2020) or not (Secemsky et al., 2019; Dinh et al., 2020; Ipema et al., 2020; Katsanos et al., 2020; Nordanstig et al., 2020) an increased risk of all-cause mortality following application of paclitaxel‐coated balloons and stents in the femoropopliteal artery. These reports support the need to develop other approaches or use other molecules. In coronary interventions, Sirolimus is now the drug of choice for DES (Byrne et al., 2017), and new devices are under evaluation to validate the use of Sirolimus-coated devices in *below the knee* peripheral arteries (Teichgraber et al., 2021). Recent studies even report the safety and efficacy of biodegradable polymer Sirolimus-eluting stent (El-Hayek et al., 2017; Pilgrim et al., 2018; Zhu et al., 2018).

Despite improved outcome with the latest generation of DES, the non-specific anti-proliferative effect of Paclitaxel and Sirolimus presents insoluble problems inherent to the nature of these molecules. As previously said, both compounds also inhibit EC proliferation, thus delaying re-endothelisation, which promotes clot formation and neo-atherosclerosis, and increases the risk of cardiovascular events. Additionally, their potent anti-proliferative effect are incompatible with systemic administration for more diffuse vascular diseases involving VSMC phenotypic switching, such as atherosclerosis.

### 3.3 New avenues of research

Numerous drugs have been tested over the years to limit restenosis, including several antiplatelet and anticoagulant drugs, calcium antagonists, lipid-lowering drugs, steroids, growth factor antagonists, and various antiproliferative agents. Since inflammation and oxidative stress have been both implied in IH, anti-inflammatory and anti-oxidant treatments were also tested to circumvent IH. Despite excellent pre-clinical results and promising initial reports, all failed to show significant effects or were abandoned due to side effects when tested in large, multicenter, randomized controlled trials. Thus, recent pre-clinical attempts using anti-inflammatory and anti-oxidant compounds to limit IH will not be discussed here. Currently, localized rapamycin-mediated inhibition of the mTOR pathway has proved beneficial *via* a numerous mechanism (see Section 3.1). Nevertheless, new avenues of research are pursued based on the latest discoveries. In this section, we highlight recent studies of the basic mechanisms that govern VSMC phenotype, which may provide new avenues to investigate for therapeutic intervention.

#### 3.3.1 Clinical potential of the gasotransmitter hydrogen sulfide (H_2_S)

Hydrogen sulfide (H_2_S) is an endogenous gasotransmitter derived from the cysteine metabolism with important vasorelaxant, cytoprotective and anti-inflammatory properties. Its vasculo-protective properties have attracted a remarkable amount of attention (Cirino et al., 2022). In this section, we review the potential clinical role of H_2_S to prevent IH.

Several studies highlighted the benefits of several H_2_S supplementation against IH *in vivo* in various models (Meng et al., 2007; Ma et al., 2012; Yang et al., 2012; Macabrey et al., 2022a; Macabrey et al., 2022b). We also showed that several H_2_S donors inhibit IH in human great saphenous vein segments *ex-vivo* (Longchamp et al., 2019; Macabrey et al., 2022a; Macabrey et al., 2022b). Recently, we demonstrated that Sodium thiosulfate (STS; Na2S2O3) works as a H_2_S donor to inhibit IH *in vivo* in a model of arterial IH, and *ex vivo* in human vein segments. Mechanistically, we showed that STS inhibits VSMC proliferation and migration *via* microtubules depolymerization (Macabrey et al., 2022b). STS is already used in the clinic to treat cyanide poisoning and to increase the solubility of calcium for the treatment of acute calciphylaxis, a rare vascular complication of patients with end-stage renal disease (Peng et al., 2018). Sodium thiosulfate is also under test in a number of clinical trials for the treatment of ectopic calcification (NCT03639779; NCT04251832; NCT02538939). STS was also tested to reduce coronary calcium in patients receiving hemodialysis (NCT00568399). Interestingly, an ongoing clinical study aims to evaluate the efficacy and safety of STS compared to placebo on myocardial infarct size in ST-segment elevation myocardial infarction (STEMI) patients treated with percutaneous coronary intervention (NCT02899364). In light of the recent proposed role of osteo-chondrogenic phenotype of VSMC in IH, the fact that STS is also used in clinical pathology to reduce calcification is of particular interest.

Given that hypertension is a major risk factor for restenosis and that Angiotensin II stimulates VSMC proliferation (see Section 2.2.4), prospective studies were conducted to test the protective effect of ACE inhibitors (ACEi) against IH. Despite initial positive results with small monocentric studies, all human trials of ACEi and angiotensin receptor blockers have been inconclusive (Langeveld et al., 2005). We recently showed that Zofenopril, an ACEi and H_2_S donor combined (Bucci et al., 2014), reduces IH in a genetic model of hypertensive mice. In addition, it suppressed IH in normotensive condition, where other non-sulfhydrylated ACEi (Enalapril, Lisinopril and Quinapril) had no effect. Furthermore, Zofenopril prevented IH in segments of human saphenous vein *ex vivo*. Mechanistically, H_2_S release from Zofenopril specifically reduced VSMC proliferation and migration *via* inhibition of the MAPK and mTOR pathways (Macabrey et al., 2022a). Further studies should be conducted to test the therapeutic potential of this particular ACEi against IH.

The use of systemic drug therapy to prevent restenosis has been almost every time unsuccessfully because of narrow therapeutic ranges, side effects and/or diminished efficacy when administered systemically (Sharma et al., 2011; Seedial et al., 2013). The focal nature of IH lesions provide a window of opportunities for the use of local drug delivery using vascular medical devices. DCB and DES, as well as peri-adventitial drug delivery have been used successfully to limit IH (Seedial et al., 2013), but strategies targeting VSMC proliferation only, while promoting endothelium recovery are needed to prevent IH. Unlike current non-specific cytostatic drugs, local H_2_S delivery might provide a unique opportunity to inhibit VSMC proliferation while promoting EC proliferation and endothelium repair. We recently developed a H_2_S-releasing biodegradable hydrogel to limit IH. This thiol-triggered hydrogel inhibited VSMC proliferation and IH in human vein segments more effectively than the sulfide salts (NaHS). Interestingly, this peptide hydrogel promoted HUVEC proliferation and transmigration *in vitro*, which may promote re-endothelisation, thereby supporting vascular repair (Longchamp et al., 2019). It was also shown that a locally applicable gel containing the H_2_S-releasing prodrug GYY4137 mitigates graft failure and improve arterial remodeling in a model of vein graft surgery in the mouse (Kip et al., 2020). We also recently demonstrated that STS, besides inhibiting VSMC proliferation and IH (Macabrey et al., 2022b), promotes EC proliferation, VEGF-induced angiogenesis and neovascularization *in vivo* (Macabrey et al., 2022c).

H_2_S works in consort with NO, and the vascular effects of NO and H_2_S are mutually supporting and entangled, with both gasotransmitter having direct and indirect effects on each other [for full review see (Cirino et al., 2022)]. All therapeutic strategies based on the use of the gasotransmitter NO have failed due to low tolerance and uncontrolled hypotensive effects (Cirino et al., 2022). It will be interesting to see whether H_2_S-based solutions can succeed where NO failed. The first challenge will be to develop stable H_2_S-donor molecules allowing slow and sustained H_2_S release over the course of months/years. Such molecules are yet to be developed and will be hard to design given the reactivity of H_2_S. Eventually, H_2_S-releasing balloons and stents could provide much-needed device to limit VSMC proliferation while promoting EC recovery.

#### 3.3.2 Targeting the YAP/TAZ-TEAD module

Emerging evidence suggest a major role of the YAP/TAZ-TEAD module is VSMC phenotype and proliferation/migration. Many molecules in development for cancer therapies inhibit YAP/TAZ/TEAD directly (Verteporfin, CA3, Super-TDU, Flufenamic acid) *via* dissociation of the YAP-TEAD interaction (Cunningham and Hansen, 2022). The YAP inhibitor verteporfin suppress YAP-induced IH in a mouse model of arterial injury (He et al., 2020) and Flufenamic acid inhibits the proliferation and migration of human aortic VSMCs *in vitro* (Schöber et al., 2002). Further experiments are necessary to evaluate further the clinical potential of these drugs against IH.

GPCR inhibitors have also been describe to inhibit YAP/TAZ-TEAD indirectly (Cunningham and Hansen, 2022). For instance, YM-254890, a specific G(α)q/11 inhibitor that indirectly inhibits YAP/TAZ (Zindel et al., 2021), inhibited IH in a mouse model of vascular injury (Kawasaki et al., 2005). However, YM-254890 also reduced systemic blood pressure and no further investigations were made in the context of IH (Kawasaki et al., 2005).

Recent study also describe a stent eluting the Sp-1 inhibitor mithramycin A, which inhibited YAP and attenuated in-stent restenosis after rabbit angioplasty (Huang et al., 2020). Prostacyclin and thromboxane A2 also regulate vasorelaxation and vasoconstriction through GPCR. Interestingly, prostacyclin analogs stimulates YAP/TAZ phosphorylation and degradation, and inhibit TEAD-dependent VSMC proliferation and migration (Kimura et al., 2016). In contrast, thromboxane A2 signaling activates YAP/TAZ to promote VSMC migration and proliferation *in vitro* (Feng et al., 2016). However, blockade of the thromboxane A2 receptor did not decrease IH in coronary angioplasty patients from the Multi-Hospital Eastern Atlantic Restenosis Trial (M-HEART II) clinical trial (Savage et al., 1995), suggesting that blocking YAP/TAZ is not sufficient to reduce IH in patients. In a recent study, Huang et al. described a Sorafenib-eluting stent, which inhibited in-stent restenosis in a rabbit carotid model, specifically through inhibition of YAP activity (Huang et al., 2019). Indeed, Sorafenib, a potent kinase inhibitor and anti-cancer molecule, seems to sequester YAP, thereby facilitating formation of the SRF/MYOCD complex and expression of VSMC-specific contractile genes (Huang et al., 2019). However, Sorafenib is a non-specific inhibitor of growth factor receptors known to impair EC proliferation and tumoral angiogenesis (Liu et al., 2006; Qi et al., 2022), which may impair endothelium repair and prolong the need for anti-thrombotic similarly to paclitaxel and Sirolimus.

Of note, other clinically established drugs probably target YAP/TAZ indirectly. Thus, the anti-diabetes drug metformin stimulate AMPK, which has been shown, in the context of cancer, to activate LATS1/2, thereby inhibiting YAP activity (Mo et al., 2015). Moreover, AMPK directly phosphorylates YAP Ser 94, a residue essential for the interaction with TEAD, thus disrupting the YAP-TEAD interaction (Mo et al., 2015). Although it has never been described to inhibit the YAP/TAZ/TEAD module in VSMC, Metformin has pleiotropic effect on VSMC (Deng et al., 2020), including inhibition of VSMC proliferation (Guo et al., 2013) and vascular calcification (Cao et al., 2013). However, conflicting results have been reported regarding the effect of metformin on IH *in vivo* in rat models of arterial injury (Lu et al., 2013; Guo et al., 2017; Deng et al., 2020). Interestingly, Metformin reduce the rate of restenosis after percutaneous coronary intervention (PCI) in diabetic patients, independently of glycemic control (Lexis et al., 2009). However, no large trials have been undertaken to specifically test the impact of metformin and other glucose lowering agents on restenosis in diabetic or non-diabetic patients. The cholesterol-lowering statins, which are potent inhibitors of IH (Mylonaki et al., 2018), also inhibit RhoA, leading to LATS1/2- and MST1/2-independent YAP phosphorylation and degradation (Sorrentino et al., 2014).

#### 3.3.3 Targeting epigenetic regulators

As mentioned earlier, epigenetic modifications of VSMC genes, especially the MYOCD gene and SRF/MYOCD target genes, play an important role in VSMC phenotypic modulations. Several studies show that HDACs are upregulated in response to growth factors and by arterial injury [reviewed in (272)]. HDAC4 contributes to PDGF-BB-induced VSMCs proliferation and migration (Usui et al., 2014). Interestingly, in that study, HDAC4 targeting using the class IIa HDAC inhibitor MC1568 decreased IH in a mouse model of carotid ligation (Usui et al., 2014). Selective inhibition of HDAC6 using tubastatin A enhanced the nuclear activity of SRF *via* increased translocation of MTRF-A, thereby preventing VSMC dedifferentiation *in vitro* and IH *in vivo* (Zhang et al., 2018). Similarly, Scriptaid, a potent pan HDAC inhibitor, decreases IH in a mouse model of arterial injury (Findeisen et al., 2011). In contrast, several studies showed that the pan HDAC inhibitor trichostatin A stimulates Akt-dependent VSMC proliferation and IH (Choi et al., 2005; Song et al., 2010; Yang et al., 2021). Obviously, further pre-clinical studies are required before testing HDAC inhibitors in human cardiovascular disease.

Another interesting strategy to reduce IH would be to increase TET2 expression. Indeed, TET2 overexpression is sufficient to induce a contractile phenotype and local viral-mediated TET2 overexpression at the site of injury in a mouse model of femoral wire injury reduced IH (Liu et al., 2013). Pharmacological avenues to induce TET2 include vitamin C, which works as a cofactor to promote TET2 activity and demethylation (Yue and Rao, 2020). The other co-factors of TET2 Fe(II) and 2-oxoglutarate, or its close metabolite *α*-ketoglutarate, could also be used to increase TET2 activity (Yue and Rao, 2020). Thus, Vitamin C, as a cofactor of TET enzymes, increases 5hmC formation and promotes DNA demethylation and probably genomic stability, in addition to its antioxidant properties (Brabson et al., 2021). Given the role of oxidative stress in IH, several clinical trials have assessed whether ascorbic acid (vitamin C) could limit restenosis over the years. Unfortunately, these studies usually reported modest effects of vitamin C on the incidence of restenosis (Nunes et al., 1995; Tomoda et al., 1996; Yang et al., 2019). In the multivitamins and Probucol (MVP) large study, a combination of antioxidant Probucol, vitamins C and E and beta-carotene showed promising results against restenosis after angioplasty. However, Probucol was removed from the market because of concerns about its potential QT-prolongation and pro-arrhythmic effects (Tardif et al., 1997; Cote et al., 1999). That said, given that Vitamin C is inexpensive and safe, further studies should be conducted to assess its potential against restenosis. Interestingly, Vitamin C promotes EC proliferation while inhibiting VSMC proliferation (Kakade and Mani, 2013; Ceresnakova et al., 2021). This property alone warrants further investigations into the development of stents and balloon releasing Vitamin C, probably in combination with more potent VSMC inhibitors.

It was also shown that the DNA methyltransferase DNMT-1 suppress the TET2 gene in VSMCs (Zhuang et al., 2017), so DNMT-1 inhibitors could prove useful to increase TET2 activity. In that study the DNMT inhibitor 5-aza-2’-deoxycytidine reduced IH in a mouse model (Zhuang et al., 2017). However, the covalent DNMT1 inhibitors 5-azacytidine and decitabine, which are widely used in research to reduce DNA methylation levels, are rapidly cytotoxic. Thus, DNMT-1 inhibitors have limited translational potential. Interestingly, TET2 knockdown prevented rapamycin-induced VSMC differentiation, suggesting that TET2 is required for the effect of Rapamycin and thus an indirect target of Rapamycin (Liu et al., 2013). It was also recently shown that TET2 expression is under the control of non-coding RNA miR-22-3p and circMap3k5 (Zeng et al., 2021), so that non-coding RNA therapies targeting TET2 may be useful in the future. Again, the development of stents or balloons releasing epigenetic modulators able to revert the synthetic phenotype could provide new device to limit IH and restenosis.

## 4 Conclusion

As highlighted in this review, there is still a lot to learn about the mechanisms governing VSMC phenotype in native vessels and in the context of cardiovascular diseases. Recent advances in genetic tools, single cell and spatial omics allow, for the first time, to dissect the molecular signature of single cells and carry out detailed and precise analyses of VSMC dynamics in health and disease. So far, single cell RNA seq has only been used in the context of atherosclerosis with a sharp focus on immune cells, rather than VSMC. It will be interesting to see single cell RNA seq data in various model of IH. That said, single cell RNA seq is descriptive, does not allow lineage tracing, and entails loss of spatial information about the distribution of the subpopulations of cells. New techniques of spatial transcriptomic combined with multiplexed imaging (Lewis et al., 2021) are required to deepen our understanding of VSMCs in the context of IH. Such studies should be conducted in various models of IH as the origin and phenotype neointimal VSMC probably differ depending on the type of intervention, location, type and severity of injury.

VSMCs remain a target of choice for the treatment of IH and phenotypic reversal is a crucial point to prevent IH. The current approaches used to prevent IH only address VSMC proliferation, not their phenotypic identity, although both are linked closely. The main issue with current therapies is the nonspecific effect of paclitaxel and Sirolimus on cell proliferation, which requires targeted delivery and has deleterious effects on the endothelium. Moreover, DES are efficient in coronary arteries, but perform poorly in the peripheral vasculature. Therefore, other VSMC-targeted approaches are required. Understanding the spatiotemporal regulation of VSMC fate, clonality, differentiation, and phenotypic modulation will reveal mechanisms essential to discovering novel therapeutic candidates. Continued efforts on multiple fronts are necessary to translate these targets into viable cardiovascular therapies. Given the complexity of disease presentation and comorbidities, combining local delivery and systemic oral drug administration will likely be necessary to treat IH, as well as diffuse vascular pathologies such as atherosclerosis and vascular calcification. Another challenge for either systemic or local release reside in the delivery system. The development of DCB and DES releasing paclitaxel or Sirolimus led to innovative delivery systems. Gels, nanoparticles, multiple-layer coatings and biodegradable scaffolds are being developed to allow sustained drug release. It will be interesting to combine these delivery systems with new molecules.
